# High-plex imaging and cellular neighborhood spatial analysis reveals multiple immune escape and suppression patterns in diffuse large B-cell lymphoma

**DOI:** 10.1038/s41375-024-02239-1

**Published:** 2024-04-04

**Authors:** David J. Reiss, Yumi Nakayama, Andrew P. Weng, Matthew E. Stokes, Laurie Sehn, Christian Steidl, David W. Scott, C. Chris Huang, Anita K. Gandhi

**Affiliations:** 1 Informatics and Predictive Sciences, Bristol Myers Squibb, Seattle, WA USA; 2 Translational Medicine Hematology, Bristol Myers Squibb, Summit, NJ USA; 3 Terry Fox Lab, BC Cancer, Vancouver, BC Canada; 4 Informatics and Predictive Sciences, Bristol Myers Squibb, Summit, NJ USA; 5 Centre for Lymphoid Cancer, BC Cancer, Vancouver, BC Canada

**Keywords:** B-cell lymphoma, Cancer microenvironment

## To the Editor:

A hallmark of hematological malignancies such as diffuse large B-cell lymphoma (DLBCL) is heterogeneity, with contribution from the tumor and its microenvironment. Application of next generation sequencing technology to patient tumor biopsies revealed not only genetic and epigenetic underpinnings of tumor intrinsic heterogeneity [1, 2], but also the complexities of the tumor microenvironment (TME). Furthermore, the lymphoma microenvironment has been characterized through computational inference or digital cytometry techniques from large collections of DLBCL gene expression data [3, 4]. These analyses demonstrated that despite lacking a clear structure of tumor/immune compartments found in solid tumors, the lymphoma TME is not a random assortment of tumor and immune cells. We believe the spatial relationship between tumor and infiltrating immune cells is a missing piece in our understanding.

**Fig. 1:**
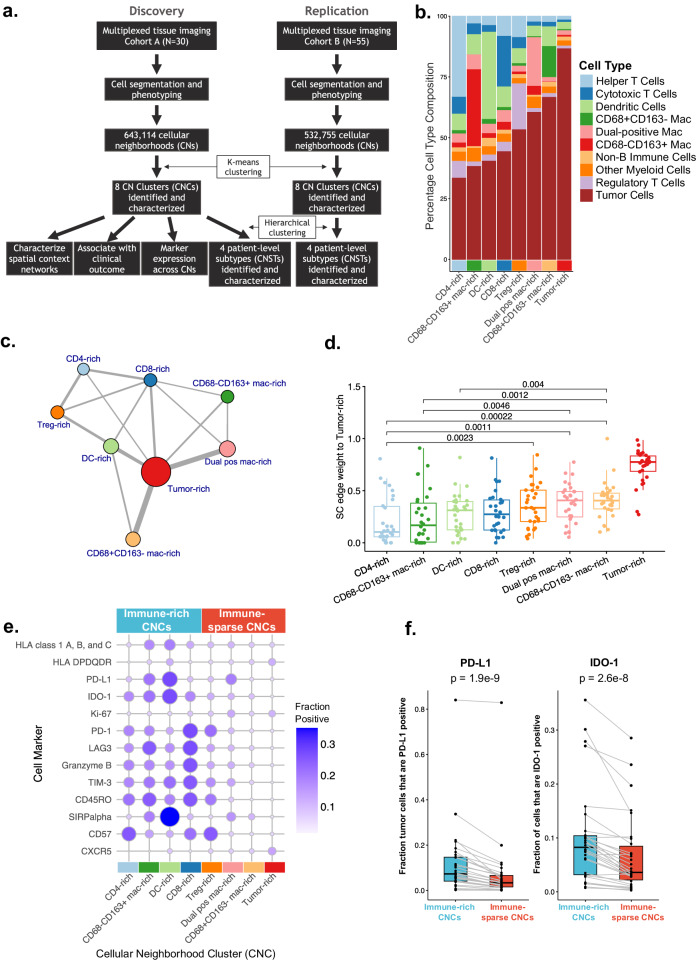
Study overview and spatial characterization of cellular neighborhood clusters (CNCs). **a** Study design and analysis flow. **b** Cell type composition of each cellular neighborhood cluster (CNC). The CNCs are ordered by increasing tumor content. **c** Aggregate spatial context networks across all samples in Cohort A. Each CNC is represented as a colored node, with node sizes proportional to the total number of CNCs in the samples. Edges between two CNCs are shown if >10% of CNs within the two CNCs were in close proximity. Edge thickness (weight) is proportional to this percentage of close proximity between pairs of CNCs. Self-edges are not included for visual clarity. **d** Spatial context network edge weights (fraction of CNs within each CNC in close proximity) between tumor-rich CNs and all other CNs, for each sample in Cohort A. This is a quantified depiction of the edge weights shown in **c**, between tumor-rich CNs and all other CNs. **e** Relative expression of functional markers across all eight CNCs, for Cohort A. CNCs are ranked based on immune cell abundance and labeled as immune-rich and immune-sparse. **f** The expression of PD-L1 or IDO-1 on tumor cells within each sample. *p* values from Wilcoxon paired test.

Technological advances in quantitative high-plex imaging such as multiplexed ion beam imaging (MIBI) and imaging mass cytometry (IMC) have provided not only quantitative measurement of protein markers but also high-resolution images to reveal spatial relations among tumor and infiltrated non-tumor cells, complementing bulk transcriptomic data. An early example of such study assessed 30 protein markers in 33 DLBCL cases by IMC [5] and found nine cellular neighborhoods (CNs). However, detailed characterization of these CNs was lacking. Using MIBI data in a DLBCL cohort, Wright et al. recently reported identification of six cell neighborhood types and three aggregate tumor-immune microenvironments [6]. Here, we extend upon this approach and systematically characterize the spatial patterns of tumor and major infiltrating immune cells in newly diagnosed DLBCL using MIBI coupled with quantitative imaging analysis to provide a detailed characterization of these CNs, describe their spatial networks and link to clinical outcomes (Fig. 1a).

We generated MIBI data for two DLBCL cohorts: Cohort A consists of 27 newly diagnosed and 3 relapsed cases from a clinical study at BC Cancer, and patients were selected for equal numbers of achievers and non-achievers of 24-month progression-free survival (PFS). PFS data following R-CHOP treatment were available, along with baseline disease characteristics, and FISH for *MYC, BCL2*, and *BCL6* rearrangement (Table S1). Three regions-of-interest (ROIs) were manually selected from whole section by certified pathologists for each sample and assayed with a 33-marker panel (Table S2). ROI selection was aimed at diversity within tumorous tissue rather than simply tumor-rich areas. Similarly, 3–5 ROIs were selected for Cohort B, which consists of 55 newly diagnosed real-world cases with no clinical data and assayed on a 17-marker MIBI panel (Table S3). Both cohorts had RNAseq data, enabling gene expression based molecular classification [7]. We describe our findings primarily in Cohort A but also highlight observations consistent between the two cohorts.

The MIBI image analysis and quantitation, performed by IonPath Inc., (Menlo Park, CA), identified 31 unique cell phenotypes in Cohort A. Next, largely following methods in Bhate et al. [8], we calculated CNs consisting of each cell and its 20 nearest neighbors, for nine relatively abundant immune cell types (Table S4) plus tumor cells, resulting in a total of 643,114 and 532,755 CNs in the two cohorts, respectively.

**Fig. 2:**
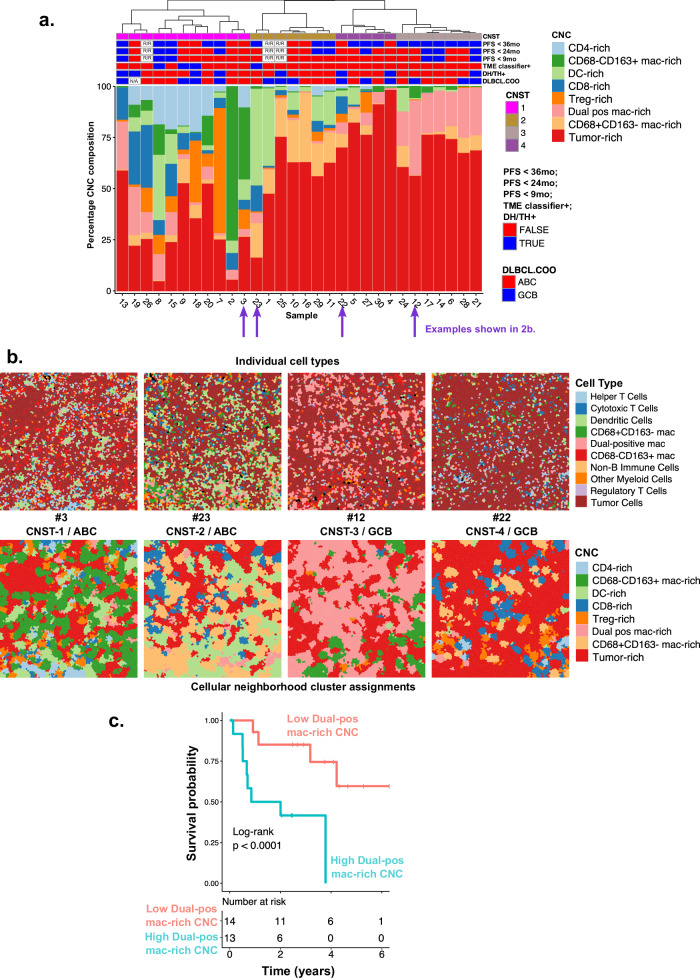
CN sample level clustering and clinical association. **a** CNC composition by patient sample for Cohort A. Hierarchical clustering of the CNC composition separated samples into four “subtypes” or CNSTs (top stacked bars). The next three stacked bars annotate these samples by their progression-free survival status at 36, 24, and 9 months; followed by tumor microenvironment signature status (TME-classifier); double-hit/triple-hit (DH/TH) status by FISH and DLBCL COO status by NanoString. R/R relapse samples, N/A undetermined. **b** Visualization of ROIs from four samples from the four CNSTs highlighted in Fig. 2a. Top: locations of individual (primary) cell types. Bottom: individual cells colored by their CNC assignment. **c** Kaplan–Meier survival plot based on PFS for dual pos mac-rich CNC by median split, log-rank test *p* value is shown.

K-means clustering of the CN cellular compositions yielded eight unique CNCs that were notably consistent between both cohorts (Fig. 1b and Fig. S1). The CNs within each CNC are enriched with different types of infiltrating immune cells, which we named with ascending tumor content as: CD4-rich (33% tumor), CD68−CD163+ mac-rich (38%), DC-rich (40%), CD8-rich (44%), Treg-rich (53%), Dual-positive (CD68+CD163+) mac-rich (60%), CD68+CD163− mac-rich (67%), and tumor-rich (87%). Exemplary MIBI ROIs colored by their CNC composition are visualized in Fig. 2b and Fig. S2.

By spatial context analysis, which measures proximities between CNs, we found that CNs within the CD4-rich CNC are the most segregated from those in the tumor-rich CNC (Fig. 1c, d). Meanwhile, putatively suppressive CNs including those in the Treg-rich and dual-positive mac-rich CNCs are more spatially proximal to the CNs in the tumor-rich CNC (Fig. 1c, d). This spatial structure, which is also suggested by the variable tumor/immune cell mixing across CNCs as shown in Fig. 1b, suggests that DLBCL tumor B cells recruit or otherwise are surrounded by a suppressive immune microenvironment.

We also found immune composition differences between the two DLBCL cell-of-origin (COO) subtypes. Relative to GCB subtype samples, ABC subtype samples are more enriched for CD68−CD163+ mac-rich CNCs (*p* = 0.008), while GCB samples have fewer immune-rich CNCs and more immune-sparse CNCs, which is in line with previously published data [9]. Additionally, when contrasting spatial context between ABC vs. GCB subtype, we find that the CD8-rich CNCs in ABC are more proximal to the CD68−CD163+ mac-rich (*p* = 0.002) and DC-rich (*p* = 0.02) CNCs (Fig. S3a and Fig. S3b). As we describe below, these two latter CNCs are more likely to be PD-L1 positive (Fig. 1e) and thus to be immunosuppressive.

These findings motivated us to examine cell marker expression patterns among the CNCs. We observed CNC-specific expression patterns of certain functional markers (Fig. 1e). For example, expression of immune suppressive ligands PD-L1 and IDO-1 was higher in general in the four immune-rich CNCs than in the four immune-sparse CNCs, particularly within the DC-rich CNCs. The CNC-specific expression of these immune suppressive markers is also associated with the expression of T-cell exhaustion and/or activation markers (PD-1, LAG3, TIM3, GZMB) (Fig. 1e), indicative of an immune suppression phenotype. Tumor cells also express these markers at significantly higher levels in the four immune-rich CNCs compared to immune-sparse CNCs (*p* < 3e-8) (Fig. 1f). This finding suggests that immune suppression by tumor cells may be induced when they are adjacent to certain immune cells, in contrast to when they are within tumor-dense regions.

Each sample varies in its CNC composition, and hierarchical clustering of sample-wise CNC composition results in four primary patient subtypes (CNSTs) (Fig. 2a). This analysis revealed several distinct DLBCL tumor/immune landscapes: a highly immune-rich subtype characterized by high T-cell-rich CNCs and CD68−CD163+ macrophage-rich CNCs (CNST-1); a tumor-mixed subtype characterized by high dendritic cell-rich and CD68+CD163− macrophage-rich CNCs (CNST-2); a tumor-rich subtype which also has high dual-positive macrophage-rich CNCs (CNST-3); and a subtype dominated by tumor-rich CNC (CNST-4). Using the same methods, we obtained four similar CNSTs in Cohort B (Fig. S4), albeit the relative proportion of these four CNSTs varies between the two cohorts. Example ROIs from samples selected from each CNST are illustrated in Fig. 2b.

We investigated whether CNCs and/or CNSTs are associated with high-risk molecular signatures or clinical outcome in Cohort A. The dual-positive macrophage CNC is significantly associated with shorter PFS (log-rank *p* = 0.004) (Fig. 2c and Fig. S5). Correspondingly, CNST-3, which is enriched with high dual-positive macrophage-rich CNC is associated with shorter PFS (PFS ≤ 9 months [enrichment *p* = 0.02], Fig. 2a). COO subtype and double-hit/triple-hit by FISH did not correlate significantly with CNC or with CNST. As expected, TME classifier-positive (immune-high) cases [7] are enriched in immune-rich CNST-1 (enrichment *p* = 0.01).

Our work bears similarities to a recent publication applying MIBI analysis to DLBCL [6], including identification of dendritic cell- and macrophage-enriched CNs, indicative of the strength of these analyses. The robustness of our analysis is further enhanced by showing consistency across CNCs and CNSTs in two independent cohorts. This enables us to report novel details that shed light on potentially clinically relevant characteristics of the DLBCL TME.

By dividing macrophages into three distinct subpopulations based on CD68 and CD163 markers and known biology of tumor associated macrophages [10], we could assign them to different CNCs that reflect functional difference. Notably, we showed that double-positive macrophage CNs are associated with poor clinical outcome and found a patient subtype enriched with this macrophage population (CNST-3). Our result is consistent with prior reports that higher CD68+CD163+ (also known as M2-like, or suppressive macrophages) confers poor prognosis in DLBCL [10–12].

Given seven out of eight CNCs contain at least one type of infiltrating immune cell, a fundamental question is how DLBCL tumors escape immune clearance, and three mechanisms were proposed [13]. First, it is well documented that tumor cells may achieve immune escape through extensive mutations in components of MHC complex [14, 15]. Our analysis reveals an additional mechanism: tumor cells tend to remain physically isolated from T helper cells, which perform an essential role of antigen recognition. Second, tumoral tissues are often enriched with immune suppressive cells such as CD68+CD163+ macrophages and T regulatory cells, and we show that tumor cells spatially surround themselves with immune suppressive cells. Third, tumor cells can express suppressive ligands such as PD-L1 or IDO-1, canonically expressed by immune cells. We show this aberrant expression is more prominent when the tumor is surrounded by immune cells rather than other tumor cells. How DLBCL acquires these favorable spatial arrangements is probably through tumor–TME interactions and further research to detail this is needed.

In summary, by applying cutting-edge proteomic imaging techniques and cellular neighborhood analysis, we have uncovered spatial patterns that reflect dynamic interactions between DLBCL tumor and the lymphoma TME, and their association with clinical outcome. Exploration of these spatial relations may lead to new immune-oncology therapy approaches.

### Supplementary information

Supplemental Materials for High-plex imaging and cellular neighborhood spatial analysis reveals multiple immune escape and suppression patterns in Diffuse Large B Cell Lymphoma
