# Supplementary material for: High-plex imaging and cellular neighborhood spatial analysis reveals multiple immune escape and suppression patterns in diffuse large B-cell lymphoma
Source: Leukemia. 2024 Apr 4;38(5):1164–8. doi: 10.1038/s41375-024-02239-1 (PMC11073958; doi:10.1038/s41375-024-02239-1)
Supplement: Supplementary file 1 — Supplemental Materials for High-plex imaging and cellular neighborhood spatial analysis reveals multiple immune escape and suppression patterns in Diffuse Large B Cell Lymphoma [file 41375_2024_2239_MOESM1_ESM.pdf]

Supplemental Materials for

High-plex imaging and cellular neighborhood spatial analysis reveals multiple immune escape and suppression patterns in Diffuse Large B Cell Lymphoma

David J. Reiss[1], Yumi Nakayama[2], Andrew P. Weng[3], Matthew E. Stokes[4], Laurie Sehn[5], Christian Steidl[5], David W. Scott[5], C. Chris Huang[2], and Anita K. Gandhi[2]*

[1] Informatics and Predictive Sciences, Bristol Myers Squibb, Seattle WA, USA

[2] Translational Medicine Hematology, Bristol Myers Squibb, Summit NJ, USA

[3] Terry Fox Lab, BC Cancer, Vancouver, BC Canada

[4] Informatics and Predictive Sciences, Bristol Myers Squibb, Summit NJ, USA

[5]Centre for Lymphoid Cancer, BC Cancer, Vancouver, BC, Canada

* Corresponding author. Email: Anita.Gandhi@bms.com

Table of Contents

1. Supplementary Methods....2
   - 1.1 Tissue source of Cohort B....2
   - 1.2 Tissue staining and imaging....2
   - 1.3 Cell segmentation and classification....3
   - 1.4 Cellular neighborhood analysis....3
   - 1.5 Cellular neighborhood sample-wise clustering....3
   - 1.6 CNC-specific expression patterns....4
   - 1.7 Spatial context networks....4
   - 1.8 Fluorescence in situ hybridization (FISH)....4
   - 1.9 Survival analysis....4
2. Supplementary Tables....5
   - 2.1 Baseline Clinical Characteristics of Cohort A 5
   - 2.2 MIBI markers for Cohort A....6
   - 2.3 MIBI markers for Cohort B....7
   - 2.4 Cell phenotypes and relative abundances....8
3. Supplementary Figures....9
   - 3.1 Figure S1 Cellular fraction in 8 cellular neighborhood clusters (CNCs, Cohort B)...9
   - 3.2 Figure S2 CNC spatial distribution across all three ROIs per sample (Cohort B)....10
   - 3.3 Figure S3 SC network edge weights contrasted between ABC and GCB samples..11
   - 3.4 Figure S4 CNC composition across 4 sample-wise subtypes (CNST, Cohort B).......12
   - 3.5 Figure S5 Hazard ratios and association significance for each of the eight CNCs with progression-free survival (PFS) (Cohort A) 13

4. References....14

1. <u>Supplementary Methods</u>

1.1 Tissue source of Cohort B
Cohort B consists of commercially sourced newly diagnosed DLBCL patients (Avaden Biosciences, Seattle, WA). Each sample was evaluated by 3 independent pathologists.

1.2 Tissue staining and imaging
MIBI staining is performed as in (1). Similar to traditional IHC, slides were deparaffinized, rehydrated, then heated to 97C in epitope retrieval buffer. Slides were blocked with 3% donkey serum in TBS-Tween before antibody staining, then washed and fixed.

The optimal concentration of the antibodies was determined by analyzing the images and determining the concentration at which there was maximum signal in the cells of interest with minimal background. Numerous different tissue types were analyzed throughout the validation process to ensure the suggested lower limit of detection was achieved at the optimal concentration whenever possible.

The samples were stained in a single batch along with an Ionpath control TMA containing liver, placenta, thymus, and tonsil sections. Evaluation of the antibody signal across the tonsil, thymus, placenta, and liver sections verified the performance of all markers in the panel.

For both cohorts, the ROIs were manually selected by certified pathologists from the whole excisional biopsy for MIBI imaging. We aimed at diversity within tumorous tissue rather than simply the tumor-rich area.

Cohort A was stained with a 33-marker panel, including those for tumor (CD20/PAX5), CD4+ and CD8+ T cells (CD3/CD4/CD8), Tregs (FOXP3), NK cells (CD56), M1 and M2 macrophages (CD68, CD163), dendritic cells (CD11c), monocytes and MDSCs (CD14), FRCs/Fibroblasts (Vimentin, SMA, Podoplanin), naïve and proliferating T cells (CD45RO, Ki67), and additional markers including PD-1, PD-L1, LAG3, TIM3, GZMB, CXCR5, SIRPa, HLA class A, B, C (MHC I), and HLA-DPDQDR (MHC II). For each of the N=30 ND DLBCL samples, 3 fields of view (ROIs) were selected via a point and click interface using H&E images for guidance. The ROIs were confirmed using a secondary electron detector (SED) that shows a live image of the topography of the tissue. Imaging was performed with the latest version of the MIBIScope (IonPath, Palo Alto, CA) and each ROI was 800 µm by 800 µm in size and imaged at a resolution of 2048 x 2048 pixels (0.39 µm per pixel).

Cohort B was stained with a 17-marker panel, including those for tumor (CD20/PAX5), CD4+ and CD8+ T cells (CD3/CD4/CD8), Tregs (FOXP3), NK cells (CD56), M1 and M2 macrophages (CD68, CD163), and dendritic cells (CD11c), plus additional markers PD-1, PD-L1, HLA-DR. For each of the N=55 ND DLBCL tissue samples, between 3 and 5 fields of view (ROIs) were selected. Imaging was performed with the beta version of the MIBIScope (IonPath, Palo Alto, CA) and each ROI was 500 µm by 500 µm in size and imaged at a resolution of 1024 x 1024 pixels (0.49 µm per pixel).

1.3 Cell segmentation and classification.
Cell segmentation was performed by combining the nuclear dsDNA signal with cytoplasmic and membrane markers to accurately define the seed points and boundaries in tissue images. Initial cell predictions are made using a deep learning-based object-detection model that has been trained on previously segmented MIBIscope data. Boundaries were manually reviewed by visualizing nuclear, membrane, and cytoplasmic signals. As needed, cell segmentations were refined by adding, removing, or moving watershed seed points to ensure cell instances in a given image were accounted for. The result is an image in which each pixel is either assigned an integer value corresponding to a unique cell instance or 0, indicating a cell is not present. In the end, 643,114 cells were identified across 90 ROIs among the 30 samples of Cohort A, and 532,775 cells across the 204 ROIs of Cohort B.

Cell classification was performed with a custom pipeline which builds up a set of hierarchical rules based on the markers present in the panel. In appropriate cases, rules were designed to be mutually exclusive such that cells containing markers without expected coexpression could not be doubly positive, such as CD3 and CD68. Expression of markers were quantified at the single cell level using scaled (arcsinh transformed) summed intensities. Thresholds for each marker were initially determined based on the histogram distribution of intensity values across all ROIs. Refinement of the thresholds was done by visually comparing the cell classifications with the biological distributions seen in the images. This analysis resulted in 31 cell phenotypes for Cohort A and 17 cell phenotypes for Cohort B.

1.4 Cellular neighborhood analysis
Our cellular neighborhood analysis framework is a custom implementation, largely based on the methods of Bhate et al., and subsequently also described in Wright, et al. (2, 3).

We calculated cellular neighborhoods (CNs) consisting of each cell and its 20 nearest neighbors, for all cells with one of nine relatively-abundant (above 5%) immune cell types plus tumor B cells in each cohort. We applied k-means clustering to the cellular composition of all CNs (separately for each cohort). We applied the simple silhouette method (4) which clearly yielded eight as the optimal number of CN clusters (CNCs), independently for each cohort (additional model selection criteria such as AIC and BIC supported this selection).

Figure S2 visually demonstrates the CN composition across ROIs within and between samples for Cohort B (similar to Figure 2b for Cohort A).

1.5 Cellular neighborhood sample-wise clustering
We performed hierarchical clustering of the sample-wise CNC composition (distance metric: Pearson correlation, linkage: complete) and cut the clustering tree at a correlation > 0.3, to yield four sample clusters (CNSTs) in both cohorts. We then performed enrichment analysis across the various patient subsets to identify which of these four CNSTs were significantly enriched for different markers of patient risk (DLBCL COO, DH/TH, TME26, PFS).

1.6 CNC-specific expression patterns

Each CNC is a cluster of individual CNs, each of which surrounds an individual cell. We quantified the frequency with which cells express a given marker, such as PD-1 or PD-L1, above its 90% percentile of expression across all cells. These relative frequencies are depicted for all secondary markers, across all cells and all CNCs in Cohort A, in Figure 1e. Similar frequencies were computed for PD-L1 and IDO-1 expression on tumor cells only, on a per-CNC basis, aggregated across all immune-poor and immune-rich CNCs within each sample. This pairwise (within-sample) comparison is shown in Figure 1f.

1.7 Spatial context networks

Each cell was assigned to a CNC based on the cluster assignment of its CN (as visualized in Figure 2b, bottom). We identified each cell's 50 nearest neighbors and quantified the composition of CNC assignments among those 50 cells. This CNC composition quantifies a relative frequency of proximity between the cell's CNC and any neighboring CNCs. These frequencies, aggregated across either a single sample, or a subgroup of samples, or across an entire cohort, defines the edge weights between pairs of CNCs, as depicted in Figure 1c and Figure S3.

1.8 Fluorescence in situ hybridization (FISH)

FISH was performed using commercially available dual-color break-apart probes for MYC, BCL2 and BCL6 according to previously described method (5).

1.9 Survival analysis

For Cohort A, we applied Cox proportional hazards regression of the sample-wise CNC composition against progression-free survival (PFS) and computed the stratified log-rank score for each CNC when separated into two classes (high vs. low) by median threshold. No secondary covariates were included.

Table S1. Baseline Clinical Characteristics of Cohort A

The study was reviewed and approved by the University of British Columbia/BC Cancer Research Ethics Board (H18-00469, H22-03661), in accordance with the Declaration of Helsinki. Written informed consent or consent waivers were obtained for all samples utilized for research according to protocols approved by the University of British Columbia/BC Cancer Research Ethics Board.

	Median (min, max) or N (percentage)
Age	62.5 (19, 80)
Sex	
F	9 (30%)
M	21 (70%)
IPI Group	
1	8 (27%)
2	11 (36%)
3	8 (27%)
RR	NA (10%)
COO by NanoString	
ABC	15 (50%)
GCB	13 (43%)
UNC	2 (7%)
FISH	
MYC-R (incl)	13 (43%)
BCL2-R (incl)	10 (33%)
BCL6-R (incl)	7 (23%)
DH/TH	8 (27%)
TME classifier (6)	
positive	5 (17%)
negative	25 (83%)
Location	
Nodal	24 (80%)
Extranodal	6 (20%)

Table S2. MIBI Markers for Cohort A

Target	Clone
dsDNA	35I9 DNA
beta-tubulin	D3U1W
Na-K-ATPase	D4Y7E
CD57	HNK-1
CD163	10D6
CD4	EPR6855
CD11c	EP1347Y
FoxP3	236A/E7
LAG-3	17B4
PD-1	D4W2J
PD-L1	E1L3N
Granzyme B	D6E9W
CD56	MRQ-42
CD31	EP3095
Ki-67	D2H10
CD14	D7A2T
CD11b	D6X1N
CD68	D4B9C
CD8	C8/144B
CD3	MRQ-39
CD45RO	UCHL1
TIM-3	EPR22241
Vimentin	D21H3
SMA	D4K9N
PAX5	D7H5X
CD20	L26
CXCR5	D6L3C
Podoplanin	D2-40
IDO-1	EPR20374
HLA DPDQDR	C3/43
SIRPalpha	Polyclonal
CD45	2B11 & PD7/26
HLA class 1	EMR8-5

Table S3. MIBI Markers for Cohort B

Target	Clone
dsDNA	35I9 DNA
beta-tubulin	D3U1W
CD163	EPR14643-36
CD4	EPR6855
CD11c	EP1347Y
FOXP3_AF488	236A/E7
Alexa Fluor 488	Polyclonal
PD-1	D4W2J
PD-L1	E1L3N
CD56	MRQ-42
CD68	D4B9C
CD8	C8/144B
CD3	D7A6E
PAX5	D7H5X
CD20	L26
CD21	EP3093
HLA DR	EPR3692
CD45	2B11 & PD7/26
HLA class 1 A, B, and C	EMR8-5
Na-K-ATPase alpha1	D4Y7E

Table S4. Cell phenotype and relative abundance of Cohort A

Cell type	Markers to define	Relative abundance (median of % of nucleated cells)
Helper T Cell	CD3+CD4+	1.54
Cytotoxic T Cell	CD3+CD8+	2.15
Dendritic Cell	CD11c+HLA-DPDQDR+	0.89
CD68+CD163- mac	CD68+CD163-	1.23
Dual-positive mac	CD68+CD163+	3.09
CD68-CD163+ mac	CD68-CD163+	1.16
Non-B Immune Cell	CD45+, CD3+, CD8+,CD11b+, CD11c+, CD14+, CD68+, CD163+, or FOXP3+ that are not included in other cell types	0.92
Other Myeloid Cell	CD11b+ cells that are non-redundant from non-B immune	0.36
Regulatory T cell	CD3+CD4+FoxP3+	1.50
Tumor Cell	PAX5+ and/or CD20+	74.8

Figure S1. Cellular fraction in 8 cellular neighborhood clusters (CNCs, Cohort B)

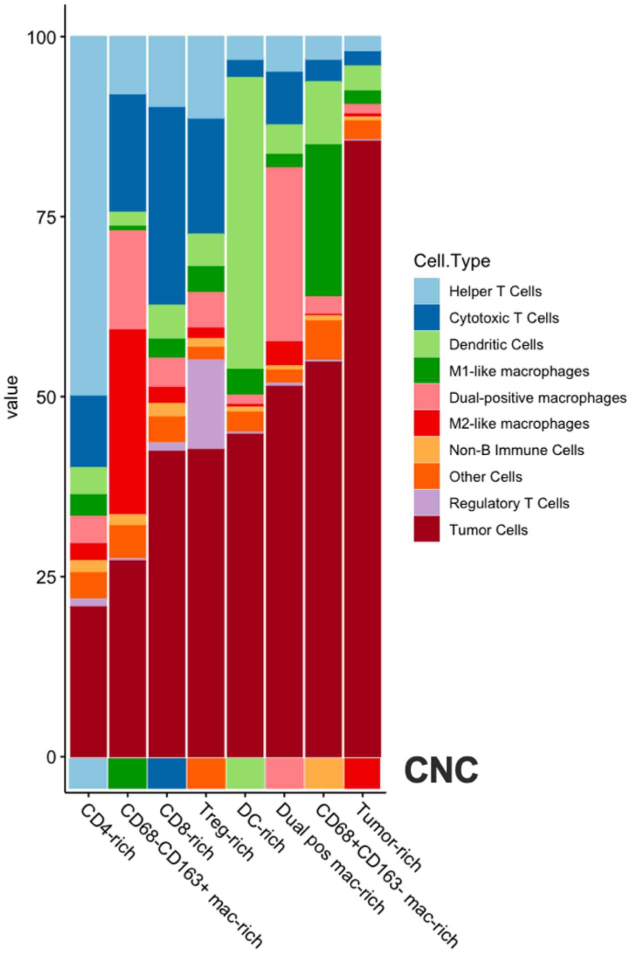

Cell type composition of each cellular neighborhood cluster (CNC), for Cohort B. See Figure 1b. for comparison with Cohort A and complete description.

Figure S2. CNC spatial distribution across three ROIs per sample

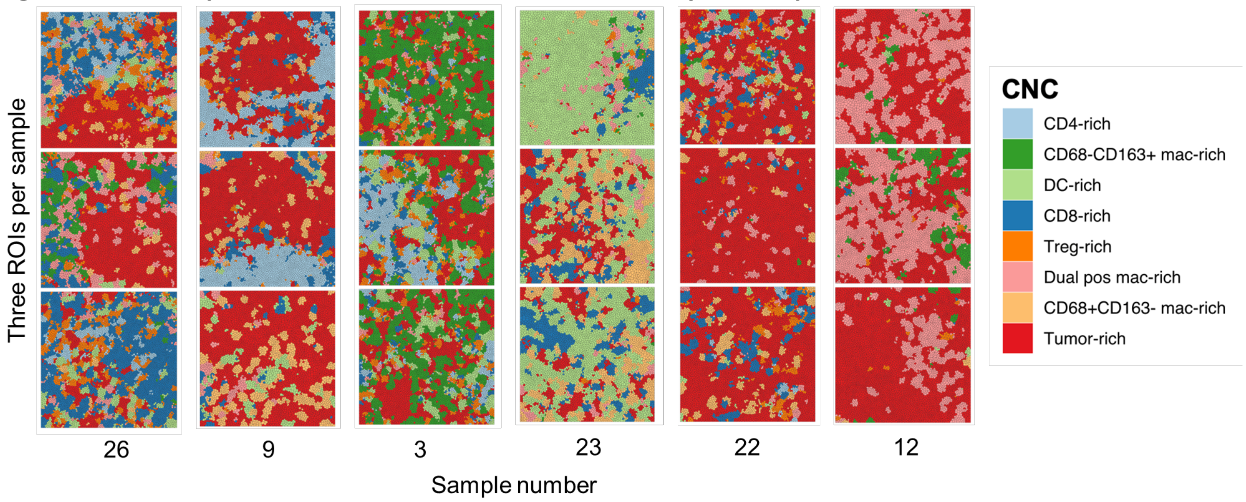

Visualization of all three ROIs from each of six randomly selected samples in Cohort A, as in the bottom of Fig. 2b.

Figure S3. Network edge weights contrasted between ABC and GCB samples

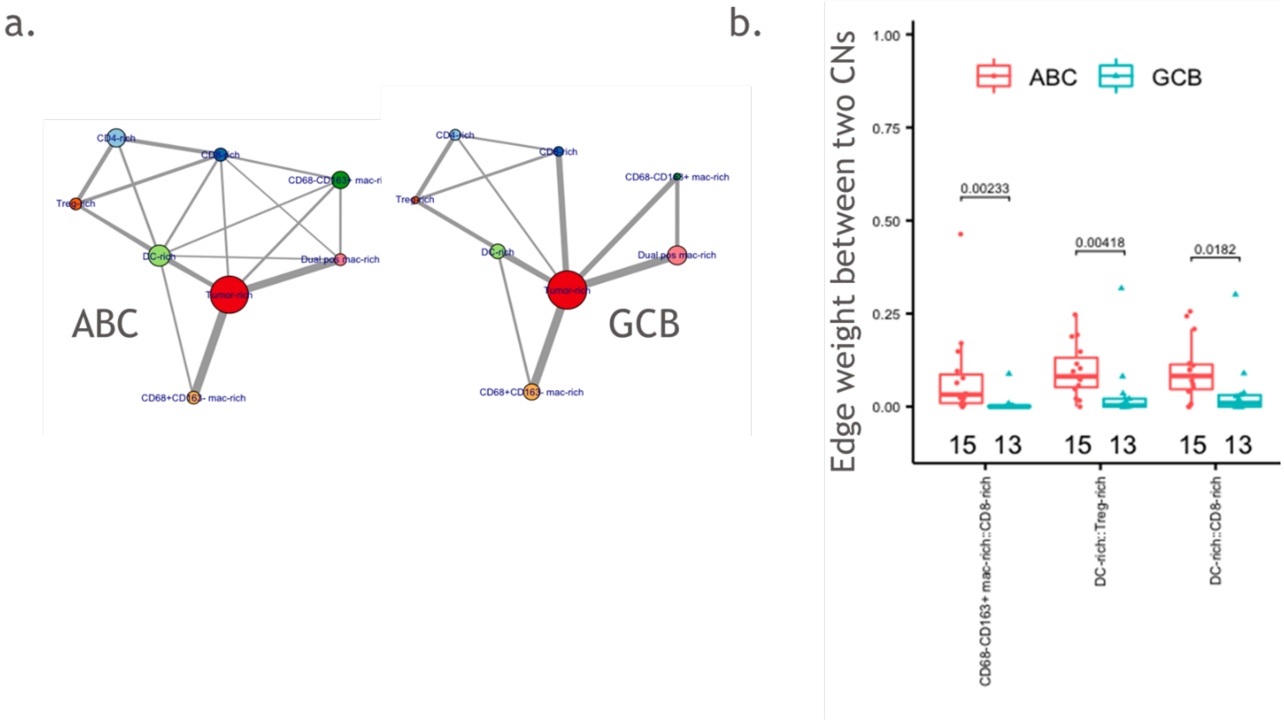

a. Spatial context networks in ABC versus GCB in Cohort A. Each CNC is represented as a colored node, with edges between two CNCs if > 10% of CNs within the two CNCs were in close proximity. Edge thickness (weight) is proportional to this percentage of close proximity between pairs of CNCs.
b. Spatial context network edge weights (as shown in a), with significant differences between ABC and GCB samples (Cohort A).

Figure S4. CNC composition across 4 sample-wise subtypes (CNSTs, Cohort B)

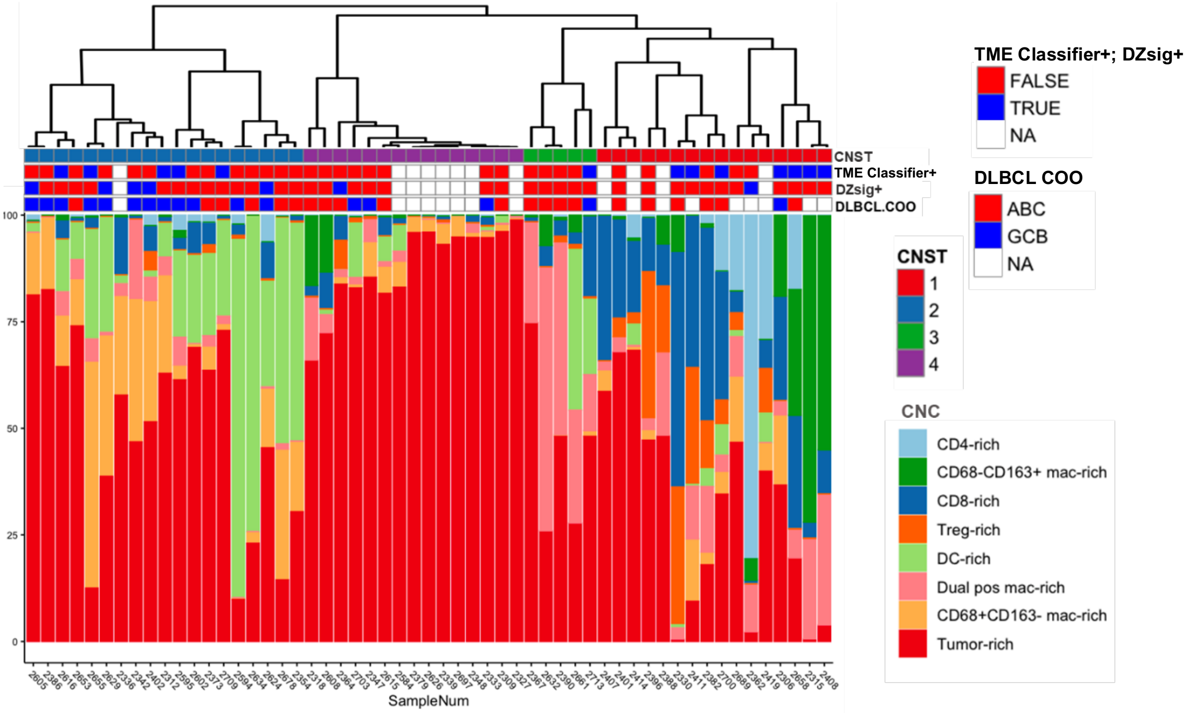

CN cluster composition by patient sample for Cohort B. Hierarchical clustering of the CNC composition separated samples into four subtypes or CNSTs (top stacked bars). The next 3 stacked bars are tumor microenvironment status (TME classifier) (6); dark-zone signature (5) and COO status by Reddy method (7). NA: undetermined (samples without RNAseq data).

Figure S5. Hazard ratios and association significance for each of the eight CNCs with progression-free survival (PFS) in Cohort A.

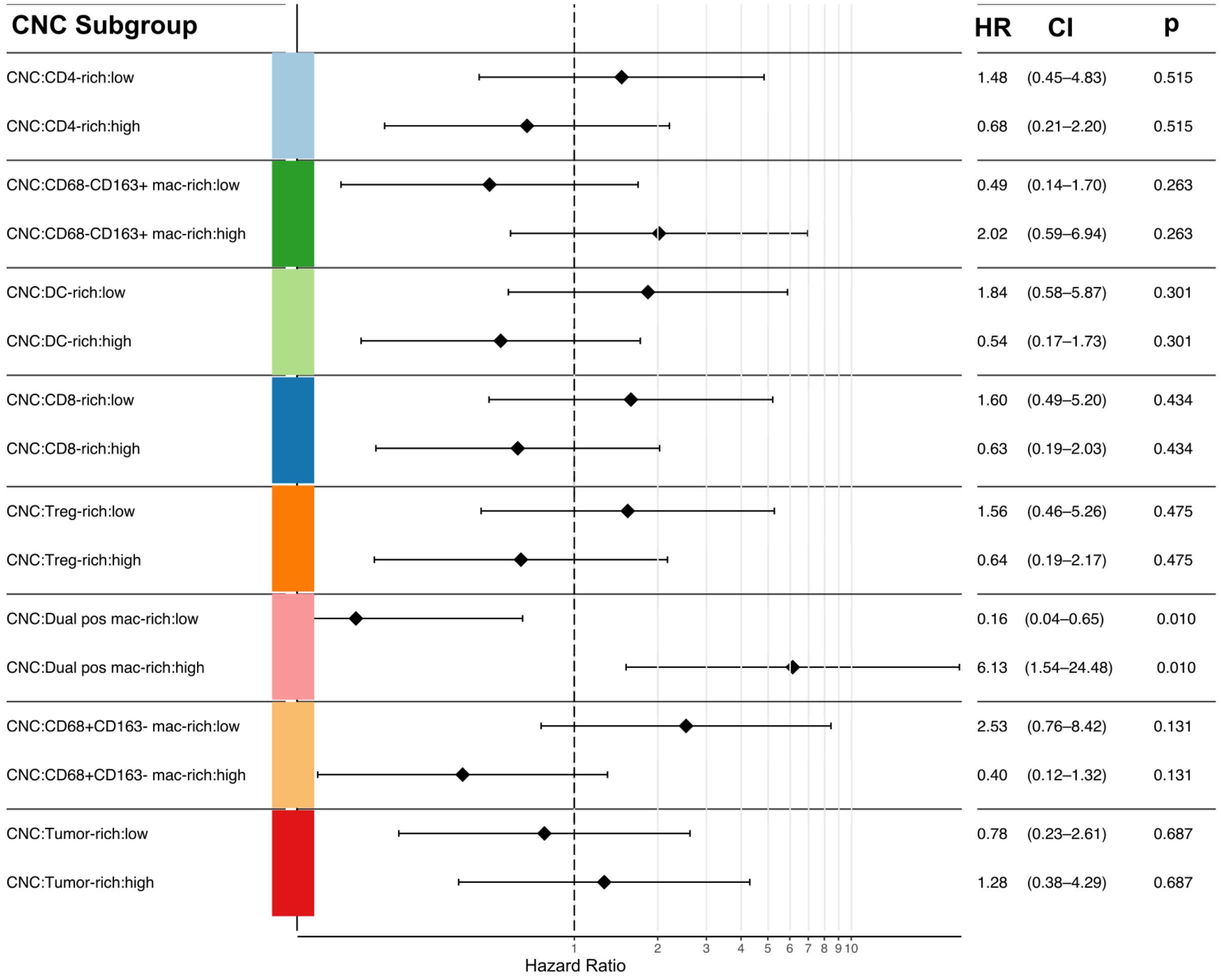

Forest plot displaying hazard ratio (high vs. low by median split) and association significance for each of the eight CNCs with progression-free survival (PFS) in Cohort A. Only the Dual pos mac-rich CNC is significantly associated with (shorter) PFS, while other CNCs including CD4-rich, CD8-rich, and CD68+CD163- mac-rich show a trend of association with (longer) PFS.

References:

1. Ptacek J, Locke D, Finck R, Cvijic ME, Li Z, Tarolli JG, et al. Multiplexed ion beam imaging (MIBI) for characterization of the tumor microenvironment across tumor types. Lab Invest. 2020;100(8):1111-23.
2. Bhate SS, Barlow GL, Schurch CM, Nolan GP. Tissue schematics map the specialization of immune tissue motifs and their appropriation by tumors. Cell Syst. 2022;13(2):109-30 e6.
3. Wright K, Weirather JL, Jiang S, Kao KZ, Sigal Y, Giobbie-Hurder A, et al. Diffuse Large B-cell Lymphomas Have Spatially-Defined Tumor-Immune Microenvironments Revealed by High-Parameter Imaging. Blood Adv. 2023;7(16):4633–46.
4. Wikipedia. Silhouette (clustering) 2023 [Available from: https://en.wikipedia.org/wiki/Silhouette_(clustering).
5. Alduaij W, Collinge B, Ben-Neriah S, Jiang A, Hilton LK, Boyle M, et al. Molecular determinants of clinical outcomes in a real-world diffuse large B-cell lymphoma population. Blood. 2023;141(20):2493-507.
6. Risueno A, Hagner PR, Towfic F, Fontanillo C, Djebbari A, Parker JS, et al. Leveraging gene expression subgroups to classify DLBCL patients and select for clinical benefit from a novel agent. Blood. 2020;135(13):1008-18.
7. Reddy A, Zhang J, Davis NS, Moffitt AB, Love CL, Waldrop A, et al. Genetic and Functional Drivers of Diffuse Large B Cell Lymphoma. Cell. 2017;171(2):481-94 e15.
